# Potential Use of Amla (*Phyllanthus emblica* L.) Fruit Extract to Protect Skin Keratinocytes from Inflammation and Apoptosis after UVB Irradiation

**DOI:** 10.3390/antiox10050703

**Published:** 2021-04-29

**Authors:** Khwandow Kunchana, Wattanased Jarisarapurin, Linda Chularojmontri, Suvara K. Wattanapitayakul

**Affiliations:** 1 Department of Pharmacology, Faculty of Medicine, Srinakharinwirot University, Bangkok 10110, Thailand; Khwandow.kun@g.swu.ac.th (K.K.); wattanased.jarisalapurin@g.swu.ac.th (W.J.); 2 Department of Preclinical Sciences, Faculty of Medicine, Thammasat University, Khlong Luang, Pathum Thani 12121, Thailand; clinda@staff.tu.ac.th

**Keywords:** amla, *Phyllanthus emblica*, *Emblica officinalis*, keratinocytes, HaCaT, ultraviolet rays, UVB, oxidative stress, reactive oxygen species (ROS), antioxidants

## Abstract

Ultraviolet B (UVB) exposure is the primary risk factor for the deadliest type of skin cancer—melanoma. Incorporating natural antioxidants in skin protection products is currently a favored research theme. For this study, we selected *Phyllanthus emblica* L. fruit extract (PE) to assess its potential use in dermal protection against UVB-induced keratinocyte inflammation and apoptosis. High-performance liquid chromatography (HPLC) was used to investigate PE’s phytochemical constituents (ascorbic acid, ellagic acid, gallic acid, chlorogenic acid, and quercetin), while ferric reducing antioxidant power (FRAP), oxygen radical absorbance capacity (ORAC), total ROS, OH^•^, O_2_^•−^, and H_2_O_2_-scavenging activities were used to determine the antioxidant properties. PE significantly increased the cell viability (MTT assay) and reduced apoptosis (Hoechst staining) in HaCaT cells exposed to UVB (40 mJ/cm^2^). PE abolished oxidative stress by reducing the production of intracellular ROS, O_2_^•−^ and H_2_O_2_ production. Catalase activity (but not superoxide dismutase or glutathione peroxidase activity) was enhanced in keratinocytes incubated with PE prior to UVB exposure. Western blot analysis suggested that PE inhibited cytochrome c release and inhibited the dysregulation of PI3K/Akt without any impact on p38 activation. PE attenuated the inflammatory response to UVB irradiation by inhibiting AP-1, NF-κB, and the mediator PGE_2_. Thus, PE is a candidate with great potential for use as an active ingredient in skin care products.

## 1. Introduction

UV rays are a major cause of skin inflammation, which leads to irreversible cell damage, photoaging, and skin cancer [1]. The critical mediators associated with the mechanism of UV-induced cell damage are reactive oxygen species (ROS), which trigger the formation of inflammatory signaling cascades in skin cells, inducing the production of proinflammatory cytokines and apoptotic mediators [2]. Unlike the penetration of UVA (320–400 nm), that of UVB (280–320 nm) is limited to the epidermis layer due to the shorter wavelengths, resulting in skin burning, which is the prominent risk factor for the deadliest type of skin cancer—melanoma. To prevent the development of new cancer cells, reducing risk factors and improving protective factors are equally important [3]. 

UVB radiation can cause skin cancer by direct DNA attack, generating an excessive amount of ROS which, in turn, also cause DNA damage and lipid peroxidation as well as protein modifications and degradation [4]. These damaged molecules activate several signaling pathways that are involved in cell survival, DNA repair, aging, inflammation, and apoptosis [5,6]. 

UV-induced inflammatory processes are the primary event facilitating photocarcinogenesis through the activation of the transcription factors nuclear factor-kappa B (NF-κB), hypoxia-inducible factor-1 alpha (HIF-1α), and cyclooxygenase-2 (COX-2) as well as proinflammatory cytokines, such as TNF-α [7,8]. The cellular adaptation toward cell survival or apoptosis in response to excessive ROS depends upon the collective outcomes and balance of signaling pathways, including PI3K/Akt, mitochondrion-dependent apoptosis pathways (e.g., cytochrome c release), MAPK and NF-κB [9,10]. Thus, modifying these response pathways in relation to cell survival is crucial in protecting keratinocytes from oxidative skin damage. 

The use of UVB-preventive agents prior to developing skin cancer has been extensively investigated based on these well-established signal transduction markups. Incorporating natural antioxidants from plants and marine extracts into skin care products is becoming an important research focus for anti-aging purposes and the prevention of skin cancer [11]. Synthetic sunscreen works synergistically with natural antioxidants to maintain redox homeostasis and protect against skin cell damage from UVB radiation [12]. 

This study selected Amla or Indian gooseberry, scientifically known as *Phyllanthus emblica* L. (PE, syn. *Emblica officinalis* Gaertn.; Family *Phyllanthaceae*) because of its ethnopharmacological uses for more than 5000 years in Ayurvedic medicine originated in India [13]. The plant is cultivated and distributed to nearby countries such as Bangladesh, China, Thailand and other Southeast Asian countries. A great number of studies demonstrate health benefits of phytochemicals extracted from various parts of the plant, especially the fruits, which contain phenolics, flavonoids, tannins, and vitamins that strongly associated with the antioxidant activities, anti-inflammation, anti-diabetes, anticancer, and neuroprotective properties [14]. Although some of the pharmacological properties of PE have been studied, the dermato-protective properties of PE related to UVB-induced inflammation have not been reported. Thus, we assessed the anti-inflammatory and anti-apoptotic effects of PE during exposure to UVB radiation in the spontaneously transformed aneuploid immortal keratinocyte cell line (HaCaT), which has been extensively used as a model for in vitro studies on the UVB-induced damage of adult human keratinocytes [15].

## 2. Materials and Methods

### 2.1. Materials

Dulbecco’s modified Eagle’s medium (DMEM) without phenol red, fetal bovine serum (FBS), penicillin/streptomycin, and trypsin (2.5%) were obtained from Gibco^®^ (Thermo Fisher Scientific, Waltham, MA, USA). 3-(4,5-Dimethylthiazol-2-yl)-2,5-diphenyltetrazolium bromide (MTT) reagent was procured from Bio Basic (Amherst, NY, USA). The antioxidant standards for the HPLC analysis together with other chemicals and buffers, such as DMSO, dichloro-dihydro-fluorescein diacetate (DCFH-DA), dihydroethidium (DHE), and Purpald^®^, were obtained from Sigma-Aldrich (Merck, Darmstadt, Germany). 

The anti-mouse IgG conjugated with HRP was purchased from Invitrogen (Thermo Fisher Scientific, Waltham, MA, USA). All the antibodies for the Western blot experiments were obtained from Cell Signaling Technology^®^ (Danvers, MA, USA.). All the other reagents and chemicals were purchased from Merck^®^ (Darmstadt, Germany). The cell culture wares, including plates and flasks (polystyrene coated), were obtained from Nunc Cell Culture^®^ (Thermo Fisher Scientific, Waltham, MA, USA).

### 2.2. PE Fruit Juice Extract Preparation

PE was harvested from the same location as described in a previous study by Chularojmontri et al. [16]. The extraction was performed under semi-sterile conditions. PE fruits were washed with sterile water, and the seeds were discarded. PE fruit juice was obtained using a compact juice extractor and then filtered through sterile Whatman filter paper no. 1 (GE Healthcare, Chicago, IL, USA) three times using laboratory filtration equipment and dried to a powder by lyophilization. The PE crude extract powder was kept at −40 °C until further use. A stock solution of PE (10 mg/mL) was prepared freshly for each experiment by dissolving 10 mg of PE into 1 mL of sterile Milli-Q^®^ type 1 ultrapure water (Merck Millipore, Burlington, MA, USA) and diluting the solution with cell culture medium to 10, 50, and 100 µg/mL. 

### 2.3. Analysis of Antioxidant Compounds by High-Performance Liquid Chromatography (HPLC)

The common phytoantioxidant constituents of PE were quantitatively analyzed using a Shimadzu chromatographic system (Shimadzu, Kyoto, Japan) consisting of an LC pump (LC-20AD), autosampler (SIL-20AC HT), HPLC column oven (CTO-20A), UV–VIS photodiode array detector (SPD-M20A), system controller (CBM-20A), LC solution software, and Inertsil^®^ ODS-3 reversed-phase C18 analytical column (GL, Tokyo Sciences, Japan). To prevent the degradation of antioxidant compounds, the standard and sample solutions were precooled in an autosampler cooler at 15 °C, while the column temperature was adjusted to 40 °C with a low-pressure gradient mode. The mobile phases for analysis were acetonitrile, methanol, Milli-Q^®^ type 1 ultrapure water (Merck Millipore, Burlington, MA, USA), and 20 mM phosphate buffer, pH 2.5 (PB). The standard compounds consisting of ascorbic acid, chlorogenic acid, ellagic acid, gallic acid, phyllanthin, and quercetin were used as references for the generation of standard curves. All the standards were prepared as stock solutions at 10 mg/mL and diluted to 6.25, 12.5, 25, 50, and 100 µg/mL. 

Ascorbic acid was dissolved in HPLC-grade water, while each standard stock solution of chlorogenic acid, quercetin, and phyllanthin was dissolved and diluted in 100% methanol. A gallic acid stock solution was first prepared in 100% methanol and diluted to 12.5, 25, 50, 100, and 200 µg/mL with methanol/HPLC-grade water at a ratio of 1:1. Ellagic acid was dissolved in 0.1 M NaOH followed by serial dilutions in 50% methanol. Dry PE powder was dissolved in HPLC-grade water to prepare the stock solution (50 mg/mL) and diluted with 50% methanol for HPLC analysis. The optimization of the chromatographic conditions was described by Sawant et al. [17,18]. Briefly, the photodiode array detector wavelength was set at 190–800 nm. The determination of ascorbic acid in PE applied isocratic elution with PB at the flow rate of 0.5 mL/min, λ 243 nm, and 15-min running time. The chromatographic condition of ellagic acid determination in PE included the mobile phase consisting of 70% PB (eluent A), and 30% acetonitrile (eluent B). The isocratic elution was used for the detection at flow rate 1 mL/min, λ 253 nm, and 10-min running time. The determination of gallic acid was performed under isocratic elution at the flow rate 1.5 mL/min, λ 270 nm, and 10-min running time. The mobile phase contained 95% PB (eluent A) and 5% acetonitrile (eluent B). The sample and standard solutions of chlorogenic acid, quercetin, and phyllanthin were analyzed concomitantly using gradient elution, which included PB (eluent A) and acetonitrile (eluent B) as a mobile phase at the flow rate 1.5 mL/min, and running time 30 min. The gradient elution was performed following the condition: 0–3 min (0–10% B); 3–15 min (0–90% B); 15–21 min (90% B); 21–22 min (90–10% B); 22–30 min (10% B). The UV detection wavelength of chlorogenic acid, quercetin, and phyllanthin were set at 325 nm, 255 nm, and 280 nm, respectively. The samples and standard volume of all compounds were constantly injected at 20 µL. The retention time of standard ascorbic acid, chlorogenic acid, ellagic acid, gallic acid, phyllanthin, and quercetin were appeared at 9.796, 8.626, 4.986, 4.956, 17.187, and 12.677, respectively.

### 2.4. In Vitro Analysis of ROS-Scavenging Activities and the Antioxidant Capacities of PE

We performed a hydroxyl radical scavenging assay, superoxide anion radical scavenging assay, hydrogen peroxide scavenging assay, ferric reducing antioxidant power (FRAP) assay, and oxygen radical absorbance capacity (ORAC) assay following our previous study procedures as reported by Jarisarapurin et al. [19].

### 2.5. Cell Culture

A spontaneously immortalized aneuploid human keratinocyte cell line, HaCaT, passage number 32, was obtained from CLS Cell Lines Service GmbH (Eppelheim, Germany). The HaCaT cells were grown in DMEM supplemented with 10% FBS and 1% antibiotics (100 units/mL of penicillin/streptomycin) and incubated at 37 °C in a 5% CO_2_ incubator (NuAire, Plymouth, MN, USA). The cell culture medium was changed every 3 days, and the cells were subcultured at 80% confluency. Only early cell passages (<50) were used for the assays. 

### 2.6. Cell Treatment and UVB Irradiation

HaCaT cells were pretreated with various concentrations of PE (0, 10, 50, 100, and 1000 µg/mL) and further cultured for 6 h before UVB irradiation. The non-UV control groups were treated with cell culture medium only. Prior to UVB exposure (40 mJ/cm^2^) with the BIO SUN Ultraviolet Irradiation System (Vilber Lourmat, Marne La Vallée, France), the cells were washed twice with 4 °C cold phosphate buffer saline (PBS). Then, an adequate amount of cold PBS was layered on the cells to cover the cells, in 100-mm culture dishes. After UVB irradiation, the cells were washed again with cold PBS, and then, the PBS was replaced with fresh DMEM and the cells were incubated for 18 h for further experiments.

### 2.7. Cell Viability Assay

HaCaT cells were seeded at 2 × 10^4^ cells/well in 96-well plates and incubated overnight. After 6 h of PE incubation and UVB exposure, the cells were cultured for another 18 h. Then, MTT reagent was added into the medium at a final concentration of 0.25 mg/mL, and incubation was continued for 3 h. The medium was removed, and the remaining formazan crystals were dissolved with 100% DMSO. The cellular viability of the HaCaT cells was determined by reading the absorbance at 550 nm using a SpectraMax^®^ M2e microplate reader (Molecular Devices, San Jose, CA, USA) and calculated as the percentage of cell viability with reference to the vehicle control wells (100%).

### 2.8. Determination of Intracellular ROS Generation

HaCaT cells were grown in 60-mm culture dishes at 7 × 10^5^ cells/well. After PE treatment and UVB exposure, the cells were stained with 25 µg/mL DCFH-DA for 30 min at 37 °C in the dark [18]. The cells were collected by trypsinization, centrifuged at 300 g for 5 min, and washed twice with PBS. Then, the cell concentration was adjusted to approximately 500 cells/µL in DPBS, and the cells were analyzed for intracellular ROS fluorescence with a Guava^®^ easyCyte 8HT Benchtop Flow Cytometer (Millipore-Merck, Darmstadt, Germany). The data are shown as percentages of the relative mean fluorescence intensity.

### 2.9. Detection of Intracellular Hydrogen Peroxide Levels

An Amplex^®^ Red Hydrogen Peroxide/Peroxidase Assay Kit (Thermo Fisher Scientific, Waltham, MA, USA) was used to detect the release of H_2_O_2_ from the cells. HaCaT cells were seeded in 96-well plates and cultivated overnight. Thirty minutes after UVB irradiation, a reaction mixture containing 50 µM Amplex^®^ Red reagent and 0.1 U/mL HRP in reaction buffer was added to the culture dishes, and the reaction was incubated for 10 min. The reaction was protected from light until the end of the experiment. Finally, the cellular fluorescence intensity was measured using a microplate reader (SpectraMax^®^ M2e Multimode Microplate Readers, Molecular Devices) at the excitation/emission wavelengths of 530/590 nm.

### 2.10. Detection of Intracellular Superoxide Levels 

This assay used dihydroethidium (DHE) to detect the superoxide generated within the cells [20]. DHE is oxidized by superoxide and converted to a blue fluorescent compound in the cytosol. To initiate the experiment, HaCaT cells were grown in 96-well plates at 2 × 10^4^ cells/well. After PE pretreatment and UVB irradiation, the cells were probed with 10 µM DHE for 30 min and protected from light. The cells were washed twice with PBS and analyzed under a fluorescence microplate reader at the emission/excitation wavelengths of 488/610 nm.

### 2.11. Catalase (CAT) Activity Assay

The assay was performed as previously described with certain modifications [21]. Briefly, the cell lysates were collected and mixed with the test buffer in a 96-well plate, and 35 mM H_2_O_2_ was added to the reaction mixture, which was then incubated at room temperature for 10 min on a shaker. Next, we added 3 mg/mL of Purpald^®^ (Sigma-Aldrich, St. Louis, MO, USA) in 0.5 M KOH to each well. After the chromogenic substance was generated, potassium periodate was added to stop the reaction. The catalase activity was measured by reading the absorbance at 540 nm using a microplate reader and calculated from formaldehyde standard curves. 

### 2.12. Super Oxide Dismutase (SOD) Activity Assay

The SOD activity was determined using a SOD Assay Kit-WST (Sigma-Aldrich, St. Louis, MO, USA). Protein lysates were prepared as described by the manufacturer’s instructions. Briefly, the protein lysates were mixed with WST and enzyme working solution and incubated for 20 min at 37 °C. After incubation, the amount of water-soluble formazan dye was measured according to the absorbance at 450 nm using a microplate reader. 

### 2.13. Glutathione Peroxidase (GPx) Activity Assay

A GPx assay was performed according to the method described by Wheeler et al. with minor modifications [20]. Briefly, 100 µL of GPx assay buffer (50 mM Tris buffer, pH 7.4, and 1 mM EDTA) and 20 µL of protein lysate were added to 96-well plates. One hundred microliters of the co-substrate mixer (0.6 mg/mL NADPH, 0.4 mg/mL GSH, and 5 units/mL glutathione reductase) was then added to each well, followed by 20 µL of GPx substrate containing 15 mM cumene hydroperoxide. The GPx activity was monitored according to the absorbance at 340 nm.

### 2.14. Apoptotic Analysis by Hoechst 33,342 and Propidium Iodide (PI) Staining

After the HaCaT cells were pretreated with PE and exposed to UVB, the cells were washed twice with PBS and incubated with 1 µg/mL Hoechst 33,342 in PBS for 10 min. The cells were then washed with PBS and double-stained with 1 µg/mL PI in PBS for 10 min. After the removal of the dyes, fresh PBS was added to cover the cells, and the apoptotic/necrotic cells were detected under a fluorescence microscope. At least three randomized pictures were collected, and the apoptotic nuclei were counted using ImageJ 1.42 [22].

### 2.15. PGE_2_ Detection

A Prostaglandin E_2_ Parameter Assay Kit (R&D Systems Inc, Minneapolis, MN, USA) was used to measure the release of PGE_2_ in the cell culture supernatant. The preparation of the cell lysate and assay procedures were performed according to the manufacturer’s instructions. The PGE_2_ concentrations (pg/mL) in the samples were calculated from the standard PGE_2_ curve.

### 2.16. Western Blot Analysis

HaCaT cells were seeded in 60-mm dishes. After the cell treatments, the total cytosolic proteins were collected in RIPA lysis buffer, while the nuclear lysates were extracted by using the nuclear extraction kit No. 10009277 (Cayman Chemical, Ann Arbor, MI, USA). The protein concentrations were determined using the Bio-Rad protein assay dye reagent (Bio-Rad Laboratories Ltd., Hercules, CA, USA). The proteins of interest were separated by SDS-PAGE and transferred onto Amersham Hybond P 0.45 PVDF blotting membranes (GE Healthcare, Chicago, IL, USA) using a mini-PROTEAN Tetra system and PowerPac™ HC power supply (Bio-Rad Laboratories Ltd., Hercules, CA, USA). 

The membranes were blocked with blocking buffer (5% BSA or 5% skim milk in TBST) for 1 h before incubation with 1:1000 dilutions of primary antibodies, including those against COX-2 (#12282), cytochrome c (cyt c, #11940), NF-κB (#4764), β-actin (#3700), phospho-c-Jun (p-c-Jun) (#9164), phospho-Akt (p-Akt) (#4051), Akt (#9272), phospho-p38 (p-p38) (#9215), and p38 (#8690) (Cell Signaling Technology, Danvers, MA, USA), overnight. Then, the membranes were washed with TBST and probed with HRP-conjugated secondary antibody (1:3000) for 1 h. 

Next, the membranes were washed with TBST and probed with Amersham ECL Prime chemiluminescent detection reagent (GE Healthcare, USA). The protein bands were detected and visualized using a gel documentation system (UVITEC, Cambridge, UK) and analyzed using ImageJ 1.42 [21]. Among five different time points (0, 15, 30, 60, and 120 min), the peak protein expression after UVB irradiation and PE pretreatment was observed at 30 min for p-Akt, Akt, p-p38, and p38 and at 120 min for NF-κB, and p-c-Jun. A time-course study of COX-2 expression and cyt c release was performed at 0, 6, 12, 18, and 24 h, and 24 h was selected for detection. 

### 2.17. Statistical Analysis

All the data are reported as the mean ± SEM. The statistical tests were performed with a means comparison by one-way analysis of variance (ANOVA) with the Dunnett post hoc test.

## 3. Results

### 3.1. Quantification of Phytoantioxidant Contents in PE

**Figure 1:**
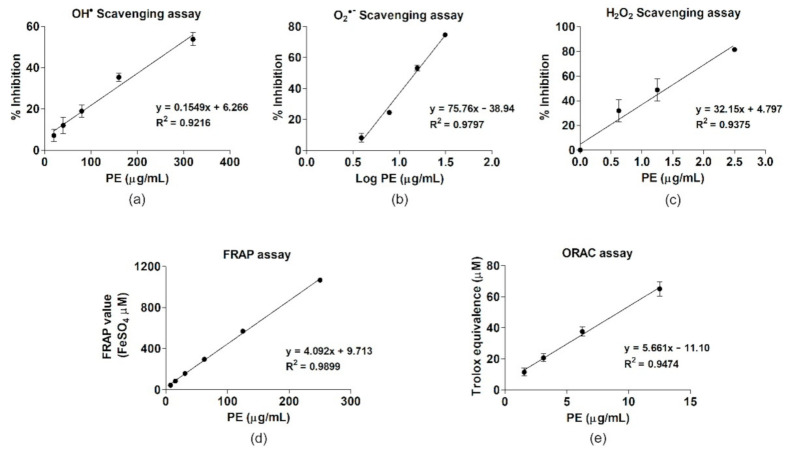
The linear regression of specific reactive oxygen species (ROS)-scavenging activities and antioxidant capacities of PE. (**a**) OH^•^, (**b**) O_2_^•−^, and (**c**) H_2_O_2_ scavenging activities. Antioxidant activities according to (**d**) FRAP and (**e**) ORAC.

The compositions of the six selected phytoantioxidants in PE were identified using HPLC to compare the specific absorption spectra and the retention times between the reference standards and samples. The retention times of the standards, including ascorbic acid, chlorogenic acid, ellagic acid, gallic acid, phyllanthin, and quercetin were 9.796, 8.626, 4.986, 4.956, 17.187, and 12.677, respectively. Shown in Figure 1 are the HPLC chromatograms of the standards (Figure S1a,c,e,g,i,k) and samples (Figure S1b,d,f,h,j,l). 

The phytoantioxidant contents were calculated from the standard calibration curves for each compound. The phytoantioxidants found in PE (% *w/w*) were ascorbic acid (1.5886%), ellagic acid (0.6255%), gallic acid (0.3702%), chlorogenic acid (0.0145%), and quercetin (0.0009%). Unexpectedly, the peak of phyllanthin was not detected in the HPLC histogram of the PE sample, although it is commonly found in the plants of the genus *Phyllanthus* (Figure S1j).

### 3.2. The Antioxidant Properties of PE

The antioxidant capacities of PE were tested in terms of specific scavenging activities (OH^•^, O_2_^•−^, and H_2_O_2_) and the total ROS-scavenging capability (FRAP and ORAC assays). Dose-dependent associations were found between the PE concentration and antioxidant activities, with significant, positive linear correlations in all the antioxidant assays (*p* < 0.05, Figure 1). The ability of PE to scavenge ROS was apparent in the concentrations of PE that inhibited half the maximal production of ROS (IC_50_, µg/mL). PE best inhibited specific ROS in the order H_2_O_2_ > O_2_^•−^ > OH^•^ (Table 1). PE also demonstrated strong antioxidant properties according to both the FRAP and ORAC assays.

### 3.3. Cytoprotective Effect of PE

The cell viability of HaCaT cells at 18 h after exposure to different doses of UVB irradiation (0, 20, 40, 60, 80, and 100 mJ/cm^2^) is shown in Figure S2a. UVB significantly induced cytotoxicity in a dose-dependent manner at doses of 40 to 1000 mJ/cm^2^. Thus, the UVB dose of 40 mJ/cm^2^, which decreased the cell viability to 75.86% ± 3.81%, was selected for the UVB treatment in further experiments. Six-hour PE pretreatment alone at concentrations up to 1000 µg/mL did not change the cell viability; however, PE at 50 µg/mL significantly increased the cell viability to 93.77% ± 4.71% in HaCaT cells exposed to UVB (Figure S2b).

### 3.4. Effects of PE on UVB-Induced ROS, O_2_^•−^, and H_2_O_2_ Production

**Figure 2:**
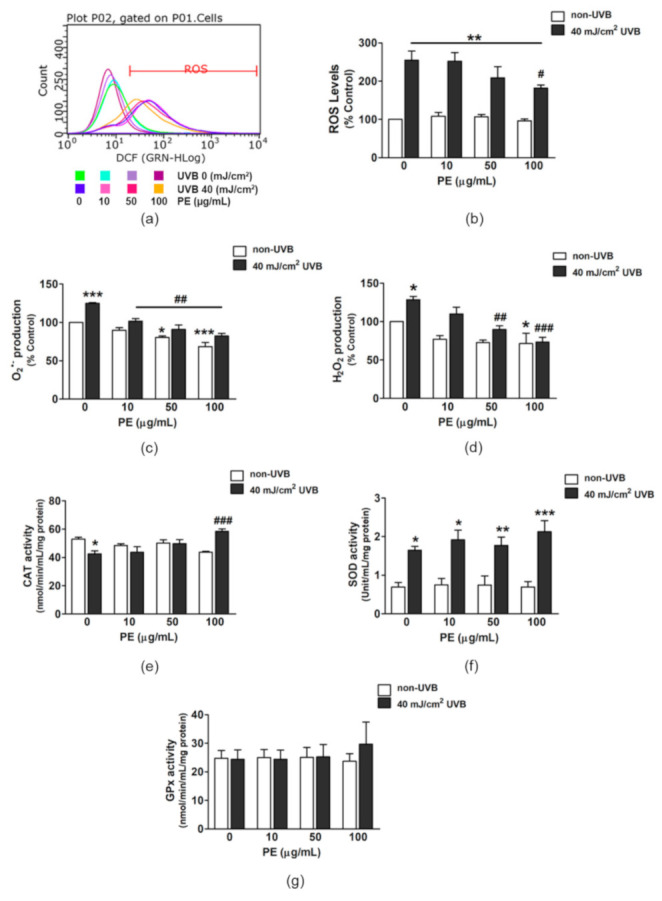
The effect of PE on UVB-induced intracellular ROS production and enzymatic antioxidant activity in HaCaT cells. (**a**) A fluorescent intensity diagram of DCFH-DA signals as detected by flow cytometry. (**b**) The calculated relative intracellular ROS levels in each sample group compared with those in the non-UVB group. (**c**) The intracellular superoxide (O_2_^•−^) production in HaCaT cells after PE preincubation and UVB exposure. (**d**) The intracellular H_2_O_2_ production in HaCaT cells following the PE incubation and UVB irradiation. (**e**) CAT activity; (**f**) SOD activity; (**g**) GPx activity. The data are shown as the mean ± SEM. *, *p* < 0.05; **, *p* < 0.01; and ***, *p* < 0.001, when compared to the non-UVB group; ^#^, *p* < 0.05; ^##^, *p* < 0.01; and ^###^, *p* < 0.001, when compared to the UVB-exposed group.

HaCaT cells exposed to UVB irradiation showed a significant 2.9-fold increase in intracellular ROS (290.76% ± 23.90%), whereas a decline of ROS levels was observed in UVB-irradiated HaCaT cells pretreated with PE (Figure 2a,b). The cells incubated with PE at 100 µg/mL before UVB exposure showed a significant attenuation of ROS production down to 181.85% ± 8.38% when compared with no PE treatment. 

UVB significantly activated the generation of O_2_^•−^ in HaCaT cells to 124.99% ± 0.89% (Figure 2c). On the contrary, PE treatment alone (10, 50, and 100 µg/mL) dose-dependently decreased cellular O_2_^•−^ accumulation down to 89.82% ± 3.37%, 80.38% ± 1.96%, and 68.50% ± 5.47%, respectively (Figure 2c), and this trend was preserved when UVB was applied to the cells. PE at 50 and 100 µg/mL significantly suppressed superoxide production down to 90.92% ± 5.80% and 82.29% ± 3.47%, respectively. 

Similarly to the generation of O_2_^•−^, the release of H_2_O_2_ in HaCaT cells was increased by UVB to 128.61% ± 8.97% (Figure 2d). After treatment with various concentrations of PE, the H_2_O_2_ production was decreased in a dose-dependent manner to 110.02% ± 12.06%, 89.85% ± 11.74%, and 73.41% ± 18.26%, for PE at 10, 50, and 100 µg/mL, respectively.

### 3.5. Effects of PE on UVB-Induced Antioxidant Enzyme Activities

The UVB-irradiated cells showed a significant decrease in CAT activity to 42.58 ± 2.12 nmol/min/mL/mg protein (80.27% ± 2.13% when compared with the non-UVB group, Figure 2e). Only PE at the highest concentration used in the experiment (100 µg/mL) significantly increased the CAT activity, to 58.46 ± 1.73 nmol/min/mL/mg protein, which represented a 30% increase from that in the UVB-exposed group (Figure 2e). In contrast to its effects on the CAT activity, UVB enhanced the SOD activity by 2.4-fold, while PE pretreatment did not affect the elevation of SOD activity (Figure 2f). The GPx activity was not changed by either UVB exposure or PE treatment (Figure 2g).

### 3.6. Effect of PE on UVB-Induced Apoptosis in HaCaT Cells

**Figure 3:**
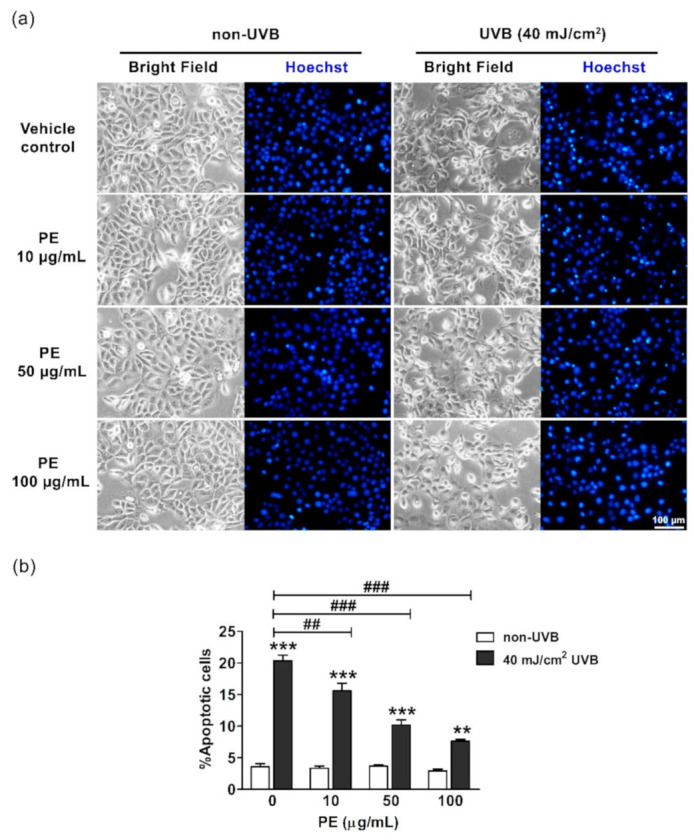
The effects of PE on UVB-induced apoptosis in HaCaT cells. (**a**) The morphological changes of HaCaT cells as observed under inverted light (bright field) and fluorescent microscopy (Hoechst staining). (**b**) The calculated percentages of apoptotic cells after PE and UVB irradiation. The data are shown as the mean ± SEM. **, *p* < 0.01, and ***, *p* < 0.001, when compared to the non-UVB group; ^##^, *p* < 0.01, and ^###^, *p* < 0.001, when compared to the UVB-exposed group. Scale bar, 100 µm.

The apoptotic morphological changes in HaCaT cells after UVB exposure included cellular shrinkage, DNA condensation, and the presence of apoptotic bodies (Figure 3a). Necrotic cell death was not detected by PI staining (data not shown). UVB irradiation significantly increased the apoptotic cells 5.7-fold, to 20.36% ± 0.87%, when compared with those in the non-UVB group, at 3.58% ± 0.49%. PE pretreatment at the concentrations of 10, 50, and 100 µg/mL significantly decreased the percentages of apoptotic cell death in a dose-dependent manner to 15.59% ± 1.21%, 10.18% ± 0.83%, and 7.65% ± 0.28%, respectively (Figure 3b).

### 3.7. Time Course Effects Regarding UVB-Induced Inflammatory and Apoptotic Signaling Pathways in HaCaT Cells

**Figure 4:**
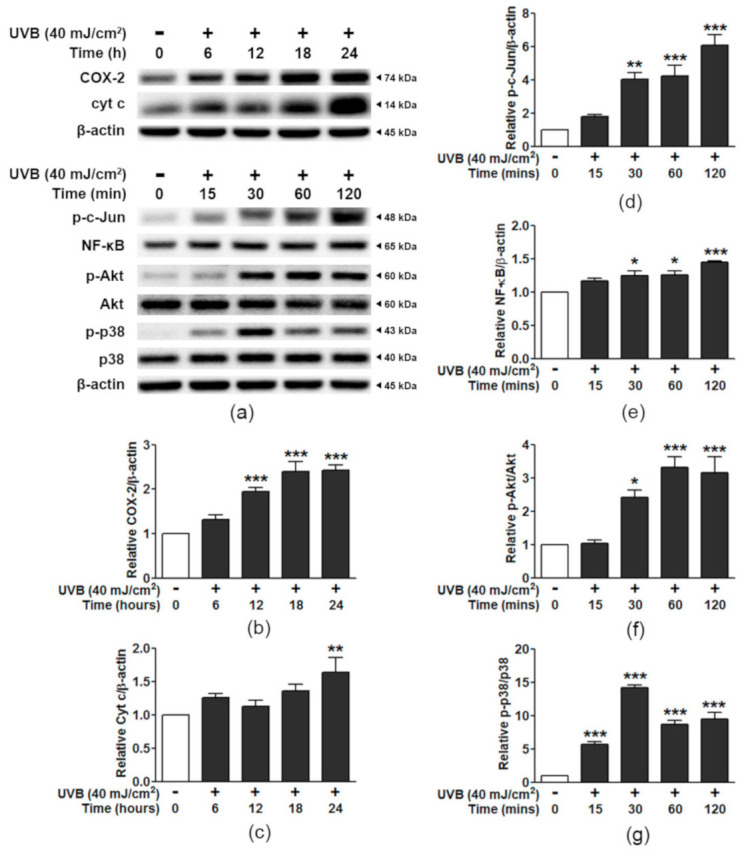
A time-course study of apoptotic and inflammatory signaling changes in HaCaT cells exposed to UVB. (**a**) Western blot bands for COX-2 expression and cyt c levels detected at 0, 6, 12, 18, and 24 h and p-c-Jun, NF-κB, Akt, and p38 detected at 0 to 120 min after UVB irradiation. (**b**) Graphical presentation of the calculated ratios of COX-2/β-actin and (**c**) cyt c/β-actin. (**d**) p-c-Jun/β-actin, (**e**) NF-κB/β-actin, (**f**) p-Akt/Akt, and (**g**) p-p38/p38. The data are shown as the mean ± SEM (*n* = 3). *, *p* < 0.05; **, *p* < 0.01; ***, *p* < 0.001, when compared to the non-UVB group.

The time-course study of the effect of UVB irradiation on keratinocyte inflammation and cell death is shown in Figure 4 and Figure S3. The UVB-activated COX-2 expression and cyt c release were found to be significantly different from non-UVB treated cells at 24 h (Figure 4a–c). The changes in signaling through c-Jun and NF-κB were the highest at 120 min (Figure 4a,d,e). For the detection of Akt and p38, the peaks of alterations occurred as early as 30 min (Figure 4a,f,g). Therefore, the effects of PE on the signal transduction in HaCaT cells exposed to UVB were studied at 24 h for COX-2 and cyt c, 120 min for c-Jun and NF-κB, and 30 min for Akt and p38.

### 3.8. Effect of PE on Inflammatory Responses to UVB

**Figure 5:**
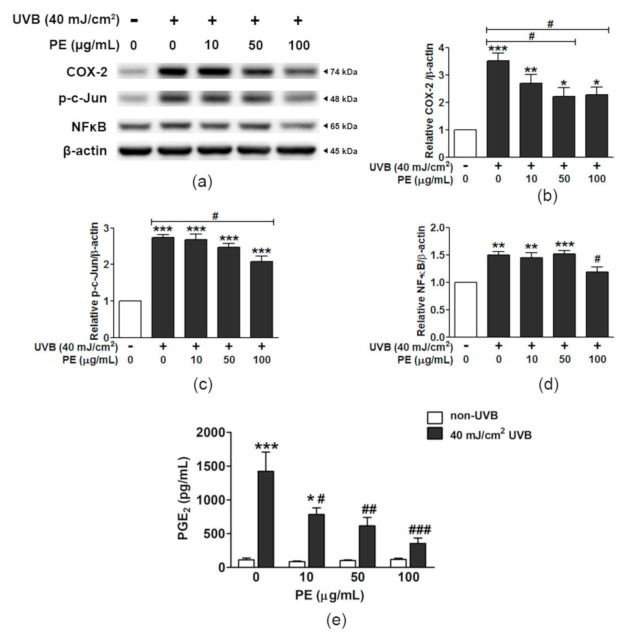
The effects of PE and UVB on the activation of COX-2, p-c-Jun, NF-κB, and Nrf2. (**a**) The protein bands of COX-2 and cyt c in relation to β-actin. The relative ratios of the band intensity for (**b**) COX-2/β-actin, (**c**) p-c-Jun/β-actin, and (**d**) NF-κB/β-actin; (**e**) the levels of prostaglandin E_2_ (PGE_2_). The data are shown as the mean ± SEM (*n* = 3). *, *p* < 0.05; **, *p* < 0.01; and ***, *p* < 0.001, when compared to the non-UVB group; ^#^, *p* < 0.05; ^##^, *p* < 0.01; and ^###^, *p* < 0.001, when compared to the UVB-exposed group.

The protective effect of PE on HaCaT cells against UVB-induced inflammation is shown in Figure 5 and  Figure S4. UVB significantly increased the COX-2/β-actin ratio 3.51 ± 0.28-fold compared to that in the non-UVB group, while pretreatment with PE markedly mitigated the effect of UVB on COX-2 activation, and, in particular, PE at the concentrations of 50 and 100 µg/mL reduced the COX-2/β-actin ratio to 2.20 ± 0.33 fold and 2.27 ± 0.27 fold, respectively (Figure 5a,b). The inflammatory nuclear signaling of c-Jun and NF-κB in HaCaT cells was also altered by UVB exposure. 

The phosphorylation of c-Jun (p-c-Jun) was increased 2.74 ± 0.07 fold by UVB; however, pretreating cells with PE at 100 µg/mL significantly decreased p-c-Jun levels, by 2.08 ± 0.16 fold, compared to those in the non-UVB group (Figure 5c). UVB increased the translocation of NF-κB by 1.50 ± 0.06-fold compared to that in the non-UVB group, whereas PE pretreatment at 100 µg/mL significantly decreased the NF-κB/β-actin ratio by 1.18 ± 0.10-fold (Figure 5d). 

The quantification of the important terminal inflammatory effector PGE_2_ is shown in Figure 5e. UVB markedly induced PGE_2_ production, up to 1423.4 ± 283.4 pg/mL (a 12.64-fold increase), compared to that in the control group (112.6 ± 26.8 pg/mL). The pretreatment of HaCaT cells with different PE concentrations (10, 50, and 100 µg/mL) significantly suppressed PGE_2_ production in a dose-dependent manner, which reduced the PGE_2_ levels down to 784.0 ± 99.2, 615.3 ± 127.1, and 355.7 ± 79.8 pg/mL, respectively. 

### 3.9. Influence of PE on Apoptotic Signaling in HaCaT Cells Exposed to UVB

**Figure 6:**
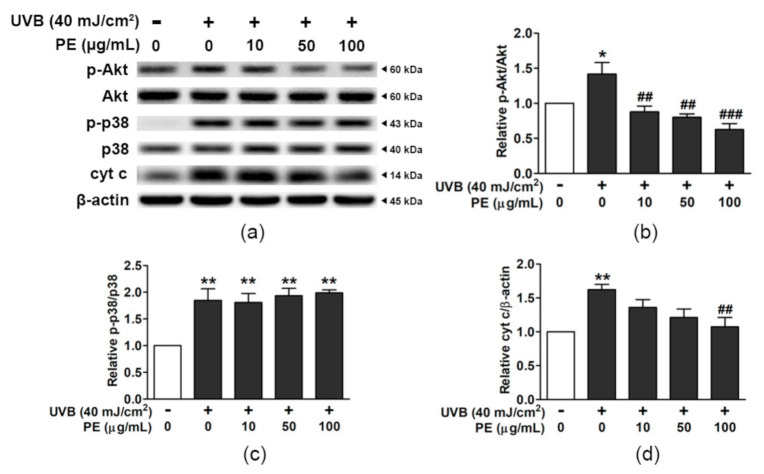
The effects of PE on the UVB-induced activation of p-Akt, p-p38, and cyt c in HaCaT cells. (**a**) The protein bands for p-Akt, Akt, p-p38, p38, cyt c, and β-actin. The relative ratios of (**b**) p-Akt/Akt, (**c**) p-p38/p38, and (**d**) cyt c/β-actin. The data are shown as the mean ± SEM (*n* = 3). *, *p* < 0.05, and ** *p* < 0.01, when compared to the non-UVB group; ^##^, *p* < 0.01, and ^###^, *p* < 0.001, when compared to the UVB-exposed group.

The cell survival and cell stress signals are presented as the relative ratios of p-Akt/Akt, p-p38/p38, and cyt c/β-actin in UVB-irradiated HaCaT cells compared to those in the non-UVB group (Figure 6 and Figure S5). UVB-exposed HaCaT cells showed enhanced phosphorylation of Akt (1.42 ± 0.17 fold); however, the pretreatment of cells with PE at 10, 50, and 100 µg/mL dose-dependently attenuated Akt activation to the levels 0.88 ± 0.08, 0.80 ± 0.05, and 0.63 ± 0.08 fold, respectively (Figure 6a,b). 

Signaling through p38 was significantly increased by 1.85 ± 0.22-fold in UVB-exposed HaCaT cells; however, PE pretreatment did not alter the triggering of p38 phosphorylation (Figure 6c). The release of cyt c in UVB-irradiated HaCaT cells increased 1.77 ± 0.12-fold; however, PE at 100 µg/mL significantly mitigated this increase in apoptotic signal down to 1.07 ± 0.14-fold (Figure 6d). 

## 4. Discussion

Repeated exposure to UVB radiation induces premature skin aging and skin cancer [23,24]. The skin epidermis, particularly the outermost layer, mainly consisting of keratinocytes, is the first layer exposed to these harmful UVB rays, leading to functional changes and cellular damage. The molecular mechanisms involved in UVB-induced keratinocyte injury include DNA damage, ROS production, inflammation, and apoptosis [25]. To prevent the development of skin cancer, avoiding excessive exposure to UV radiation or applying UV protective agents is essential. A natural PE extract has a keratoprotective effect mediated by antioxidative, anti-inflammatory, and anti-apoptotic activities in skin keratinocytes exposed to UVB irradiation.

The antioxidant effects of PE are markedly contributed to ascorbic acid, phenolics, flavonoids, and tannoids [26,27,28]. PE aqueous fruit extracts are similar in their antioxidant contents to organic solvent extracts, such as methanolic extracts [29]. Ascorbic acid is the most abundant antioxidant constituent in PE; however, there is a challenge in maintaining the stability and improving delivery to target sites when it is used for skin cosmetics and pharmaceuticals. 

Beyond their antioxidant effects, other valuable phytoantioxidants found in PE, such as chlorogenic acid, ellagic acid, gallic acid, and quercetin, could reduce skin inflammation originating from dermatitis or UV-induced oxidative damage [30,31,32,33]. Although phyllanthins are expected to be detected in plants belonging to the genus *Phyllanthus*, they were absent in the PE fruit juice extract. 

Concordant with the study by Srirama et al. [34], of 11 *Phyllanthus* species, for both methanolic and aqueous extracts, only *P. amarus* Schumach contained phyllanthin and hypophyllantin; however, these two specific phytochemicals did not reflect the hepatoprotective properties of the plant extracts. Thus, the attenuation of ROS production and the keratinoprotective effect of PE against UVB irradiation are potentially derived from other phytoantioxidants, such as the ascorbic acid, flavonoids, and phenolics found in PE. This is supported by the study by Sowa et al. [35] demonstrating that the medicinal plant extracts consisting of high antioxidant and high phenolic contents promoted skin fibroblast proliferation and wound healing. Additionally, the plant extracts from the *Carlina* genus mainly containing triterpenes and phenolic acids selectively enhanced fibroblast proliferation but inhibited UACC-903 and UACC-647 melanoma cell growth [36].

The antioxidant properties of fruit and vegetables have been widely determined through FRAP and ORAC assays [37]. The FRAP value of our PE fresh fruit extract (4.66 ± 0.27 µmol/mg) was lower than that of the PE dried fruit extract (7.46 ± 0.56 µmol/mg) previously reported from a study by Charoenteeraboon et al. [38], due to the concentration of the nutrient and antioxidant compounds in the dried form [39]. 

The measured ORAC capacity of the PE fruit juice extract (5480 μmol TE/g) was similar to the ORAC value of lemon polyphenol (5400 μmol TE/g), which exhibits very high antioxidant activity and anti-aging properties [40]. To preserve healthy skin, lemon juice extract has been used in skincare products, such as masks and skin conditioners [41]. According to the equivalent capacity of PE and lemon, PE could have skin-protective capabilities and may be used as an ingredient in skin-protection products.

A marked elevation of intracellular ROS is one of the primary phenomena observed in keratinocyte responses to UVB irradiation [42]. The specific ROS that are initially generated during UV exposure include H_2_O_2_ and O_2_^•−^, which induce overall cellular oxidative stress, leading to multiple cascades of signal transduction ultimately associated with cellular adaptations and apoptosis [43]. In vitro analysis revealed that PE juice extract was capable of scavenging important ROS generated during UV exposure, including OH^•^, O_2_^•−^, and H_2_O_2_.

PE attenuated detrimental ROS formation and scavenged the most abundant ROS that occurred during UVB irradiation. Although GPx was not altered in this study, a noticeable elevation of SOD activity in response to UVB exposure was evident in keratinocytes both with and without PE preincubation. However, the intracellular O_2_^•−^ levels were significantly reduced only in cells treated with PE. This indicates that the amount of O_2_^•−^ generated during UVB activation overwhelmed the capacity of SOD to eliminate O_2_^•−^ and maintain it at basal levels without the assistance of PE’s ROS-scavenging activity.

Keratinocytes are more vulnerable to oxidative stress than skin fibroblasts because they are lower in first-line antioxidant enzyme activities, i.e., those of SOD, CAT, and GPx [44]. Only keratinocytes overexpressing CAT showed a marked reduction in UVB-induced apoptosis; however, this protective effect was not observed in keratinocytes with SOD overexpression [45]. This suggests that H_2_O_2_ plays a critical role in keratinocyte apoptosis as induced by UVB. In this study, the highly active SOD induced by UVB generated great amounts of H_2_O_2_, which, in turn, could be removed by the action of PE through the activation of the presumably peroxidative (rather than catalytic) CAT activity, which is necessary for reducing high levels of H_2_O_2_ [46]. 

The activity of PE in scavenging H_2_O_2_ is a combination of the attenuation of intracellular H_2_O_2_ levels and a significant increase in CAT activity in keratinocytes treated with PE. PE reduced the total intracellular ROS, which partly corresponded to the decreased level of H_2_O_2_ as well as the enhanced CAT activity. The control of endogenous H_2_O_2_ is crucial, as, in aged human skin and vitiligo conditions, the accumulation of H_2_O_2_ along with a reduction in CAT activity has been observed [47,48]. 

Thus, the maintenance of the appropriate levels and harmonizing the functions of these antioxidant enzymes, rather than focusing on single enzymes, is important in balancing the cellular redox status, which works synergistically to maintain the overall defense mechanisms for cytoprotection. In high-oxidative-stress conditions, such as Down’s syndrome (DS), increased activities of SOD and GPx and reduced activity of CAT in erythrocytes are evident [49]. This imbalance may lead to detrimental effects on neuronal cells, hence impairing cognitive function in DS. 

Inflammation and apoptosis are two closely related events observed in keratinocytes exposed to UVB irradiation [50]. UV irradiation induces inflammatory signaling responses through the activation of transcription factors, including AP-1 (c-Fos/c-Jun), NF-κB, NFATs, and STATs, which, in turn, promote the production of inflammatory cytokines and prostanoids [51]. The AP-1 family protein c-Jun and the transcription factor NF-κB are the predominant UV-response genes that play important roles in the inflammatory process and the development of skin cancer [52]. PE blocked UVB-induced inflammation in HaCaT cells by suppressing AP-1 and the NF-κB–COX-2–PGE_2_ axis. 

Similar reports have shown that lutein, a natural carotenoid, reduced several proinflammatory mediators in the skin, including IL-6, TNF-α, COX-2, and matrix-metallopeptidase-9 (MMP-9) by decreasing the AP-1 signaling in UV-irradiated keratinocytes [53]. It is conceivable that quercetin, a component found in PE, may inhibit UV-induced cytokine production in HaCaT cells by suppressing the NF-κB pathway as well as inactivating the AP-1 pathway [50]. Altogether, PE inhibited key components of the inflammatory responses in HaCaT cells exposed to UVB. It is postulated that the main antioxidant components of PE such as ascorbic acid [54,55], ellagic acid [56], gallic acid [57], chlorogenic acid [32], quercetin [58], and others, are accountable for reducing proinflammatory cytokine/chemokine releases (e.g.,TNFα, IL-8, MCP-1), inhibiting signal transduction toward inflammation process (e.g., COX-2 expression and NF-κB activation), and protection against UVB-induced apoptosis.

UVB rays induce DNA damage, cell cycle arrest, and apoptosis [59]. The process of programmed cell death is typically characterized by physiological and morphological changes that depend on the stage of apoptosis. During the early stages, several events occur, including the shift of phosphatidylserine from the inner cell to the outer cell surface, the depolarization of the mitochondrial membrane potential, cell shrinkage, and chromatin condensation. In the late stages of apoptosis, the cells lose membrane integrity and fragment into apoptotic bodies [60]. 

UVB, at the dose and time of detection used in this experiment, induced late apoptosis (Hoechst staining) but not necrosis (PI staining). The intrinsic pathways that induce apoptosis were derived from the overproduction of ROS, leading to mitochondrial cyt c release, which could be inhibited by the scavenging of intracellular ROS [61]. The antioxidant property of PE inhibited the UVB-induced ROS production and reduced cyt c release, which could ultimately inhibit the triggering of downstream apoptotic executor proteins, such as caspase-9 and caspase-3. 

The molecular mechanisms of UVB-induced apoptosis also involve the tumor suppressor protein p53, which has been found to be mutated in HaCaT cells and most skin tumor cells. Blocking the mutated p53 proteins could partially block UVB-induced apoptosis in HaCaT cells [62]. Hence, other signaling pathways influence the homeostasis between cell survival and the induction of apoptosis in HaCaT cells. 

The MAPK signaling pathways, such as Akt, JNK, ERK, and p38, also play a pivotal role in the responses to UVB-induced ROS generation, leading to AP-1- and NF-κB-mediateed inflammatory processes in the skin [63], which could be deactivated by PE. The inhibition of the UV-induced activation of the Akt pathway by PE could be the mediator of the anti-inflammatory effects induced by the downstream modulation of AP-1 and NF-κB signals, as there was no alteration in p38 phosphorylation in the PE-treated keratinocytes exposed to UVB. 

**Figure 7:**
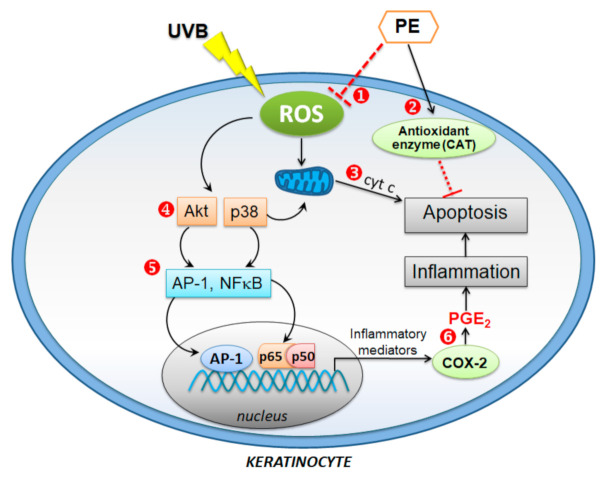
Schematic diagram depicting the proposed mechanisms of PE cytoprotection against UVB-induced HaCaT cell inflammation and apoptosis. ❶ The antioxidant effects of PE predominantly reduced ROS generation, particularly hydrogen peroxide generation, and ❷ the endogenous antioxidant defense enzyme CAT was activated. ❸ PE inhibited cyt c release. While PE had no impact on p38-induced apoptosis, it inhibited the dysregulation of PI3K/Akt ❹, which partakes in the balance of signaling pathways toward cell survival or apoptosis. PE attenuated the inflammatory response to UVB irradiation via the inhibition of the inflammatory signaling of ❺ AP-1, NF-κB, and the mediator ❻ PGE_2_.

Although a preventive role of PE on p38-induced apoptosis was excluded, p38 promoted the translocation of Bax from the cytosol to the mitochondria, causing cyt c release and caspase activation [64], maintaining that Akt signaling is crucial for cellular homeostasis and cellular adaptation promoting HaCaT cell survival and inhibiting UVB-induced cell death. Regarding this, the mechanisms of PE in protecting HaCaT cells from UVB-induced oxidative stress, inflammation, and apoptosis are summarized and demonstrated in Figure 7.

## 5. Conclusions

PE protected HaCaT cells from UVB-induced oxidative stress and apoptosis by eliminating excessive ROS, activating the antioxidant enzyme CAT, reducing cyt c release, inhibiting Akt overactivity, and mitigating the inflammatory signals AP-1 and NF-κB and the inflammatory mediator PGE_2_. The results support the use of PE as an active ingredient for skin protection from UV irradiation.

## Figures and Tables

**Table 1:** The antioxidant activities and antioxidant capacities of PE.

**ROS Scavenging Activity**	**IC_50_ (µg/mL)**	**Linear Regression Equation**
Hydroxyl radical	282.49 ± 17.59	y = 0.1549x + 6.266
Superoxide anion	14.94 ± 0.15	y = 75.46x − 38.94
Hydrogen peroxide	1.46 ± 0.37	y = 32.15x + 4.797
**Antioxidant capacity**	**(µmol/g)**	**Linear regression equation**
FRAP value (FeSO_4_ equivalent)	4279.86 ± 269.84	y = 4.092x + 9.713
ORAC (Trolox equivalent)	5480 ± 554.43	y = 5.661x − 11.10

## Data Availability

The data are contained within the article.

## Supplementary Materials

The following are available online at https://www.mdpi.com/article/10.3390/antiox10050703/s1, Figure S1: High-performance liquid chromatography (HPLC) chromatograms, standard calibration curve and DAD spectra for six phytoantioxidants in *Phyllanthus emblica* L. fruit extract (PE), including (a) ascorbic acid, (c) chlorogenic acid, (e) ellagic acid, (g) gallic acid, (i) phyllathin, and (k) quercetin. The chromatograms of the standard phyto-antioxidants are presented in the left column, while the contents in the PE sample are indicated in the right column, compared with the corresponding standards. Figure S2: The effects of UVB and PE on the HaCaT cell viability. (a) The relative cell viability of HaCaT cells after irradiation with various doses of UVB. (b) The effects of PE pretreatment followed by UVB irradiation. Figure S3: Original images of western blot band intensities in Figure 4. Figure S4: Original images of western blot band intensities in Figure 5. Figure S5: Original images of western blot band intensities in Figure 6.
