# Supplementary material for: Potential Use of Amla (Phyllanthus emblica L.) Fruit Extract to Protect Skin Keratinocytes from Inflammation and Apoptosis after UVB Irradiation
Source: Antioxidants (Basel). 2021 Apr 29;10(5):703. doi: 10.3390/antiox10050703 (PMC8146754; doi:10.3390/antiox10050703)
Supplement: Supplementary file 1 [file antioxidants-10-00703-s001.zip › antioxidants-1194562-supplementary.pdf]

Supplementary Figures

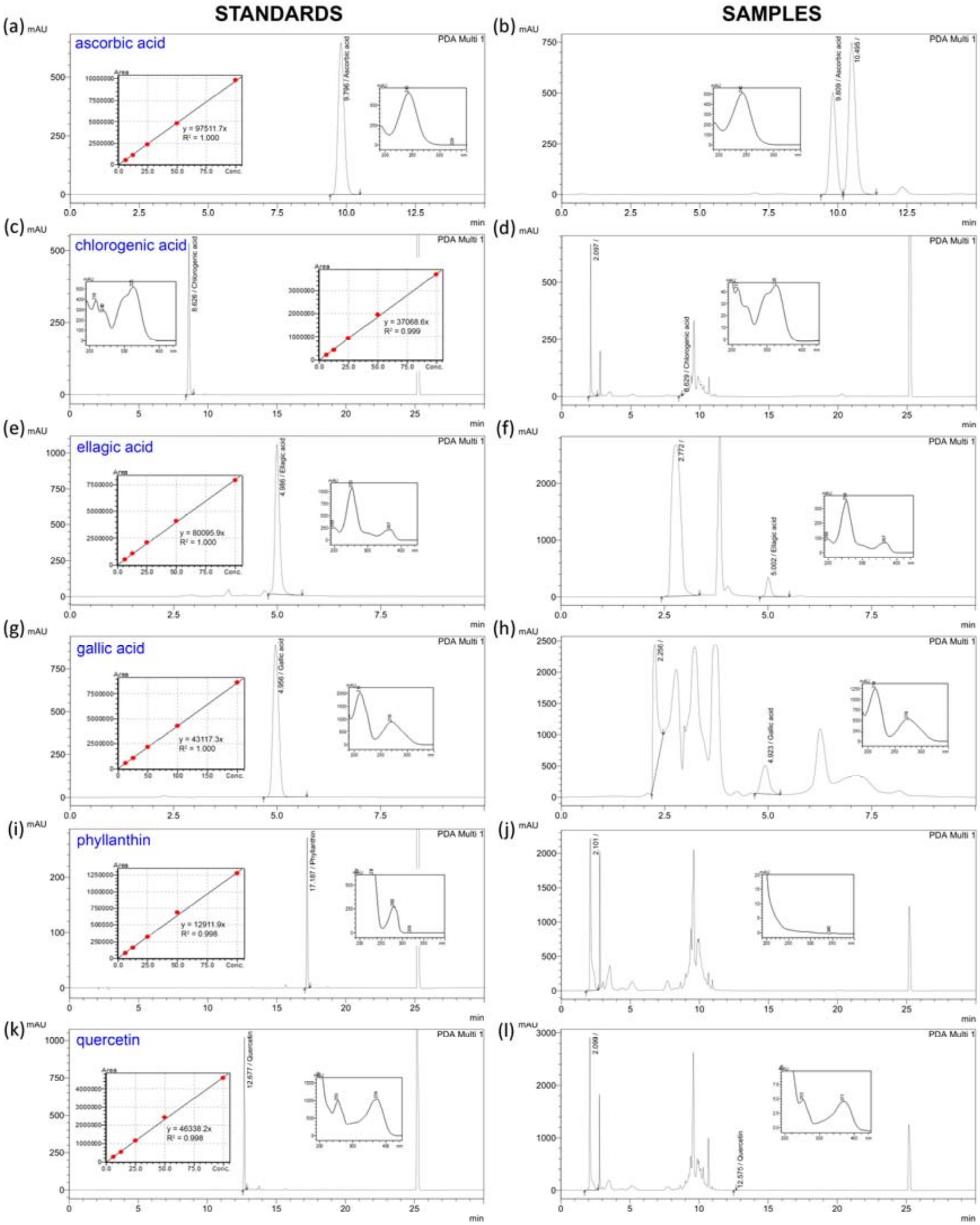

Figure S1. High-performance liquid chromatography (HPLC) chromatograms, standard calibration curve and DAD spectra for six phytoantioxidants in *Phyllanthus emblica* L. fruit extract (PE), including (a) ascorbic acid, (c) chlorogenic acid, (e) ellagic acid, (g) gallic acid, (i) phyllathin, and (k) quercetin. The chromatograms of the standard phyto-antioxidants are presented in the left column, while the contents in the PE sample are indicated in the right column, compared with the corresponding standards.

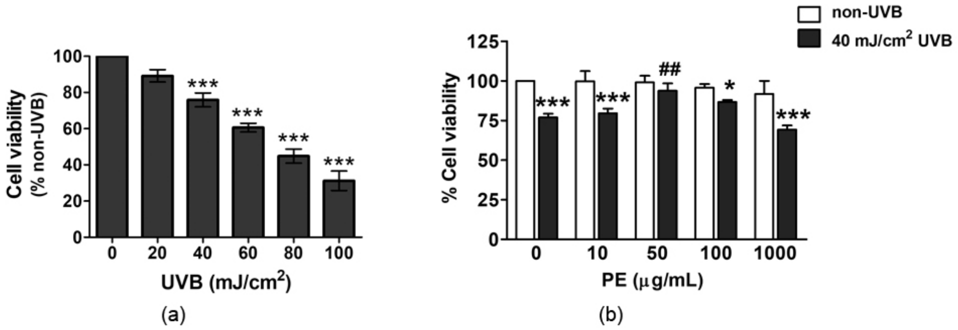

Figure S2. The effects of UVB and PE on the HaCaT cell viability. (a) The relative cell viability of HaCaT cells after irradiation with various doses of UVB. (b) The effects of PE pretreatment followed by UVB irradiation. The data are shown as the mean ± SEM. *, $p < 0.05$, and ***, $p < 0.001$, when compared to the non-UVB group; $^{\#\#}$, $p < 0.01$, when compared to the UVB-exposed group.

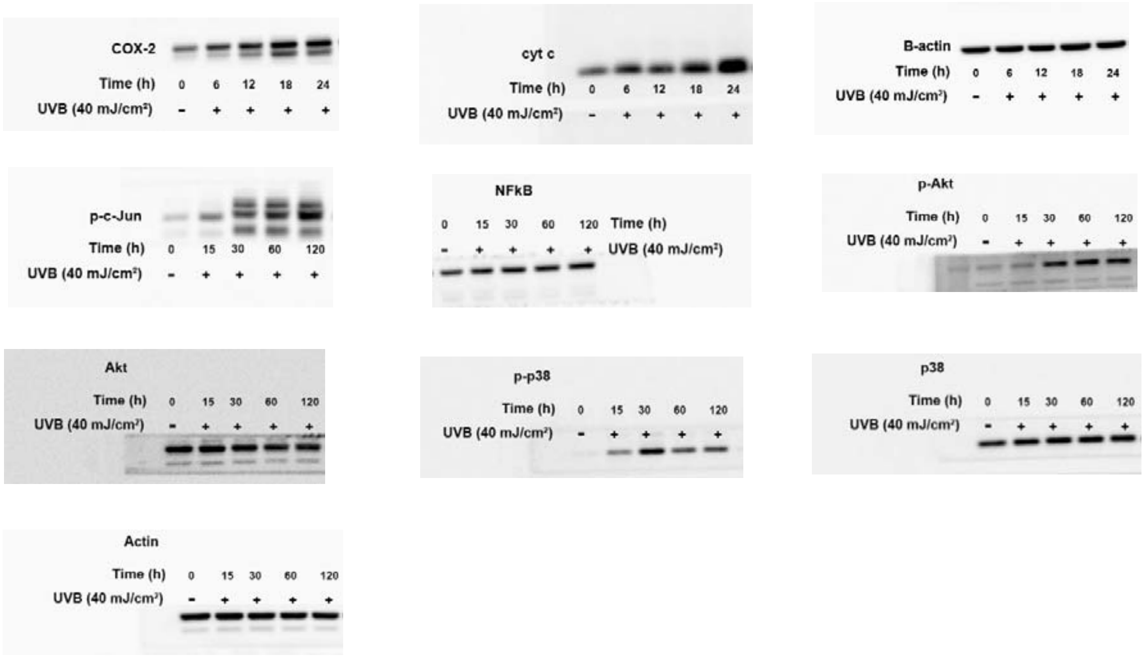

Figure S3. Original images of western blot band intensities in Figure 4.

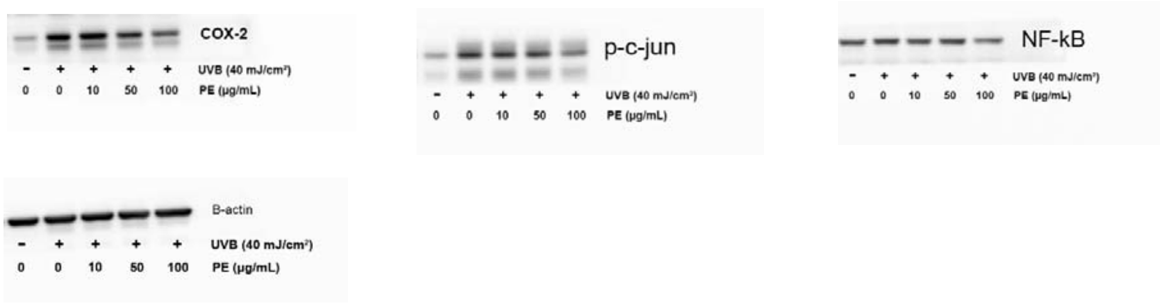

Figure S4. Original images of western blot band intensities in Figure 5.

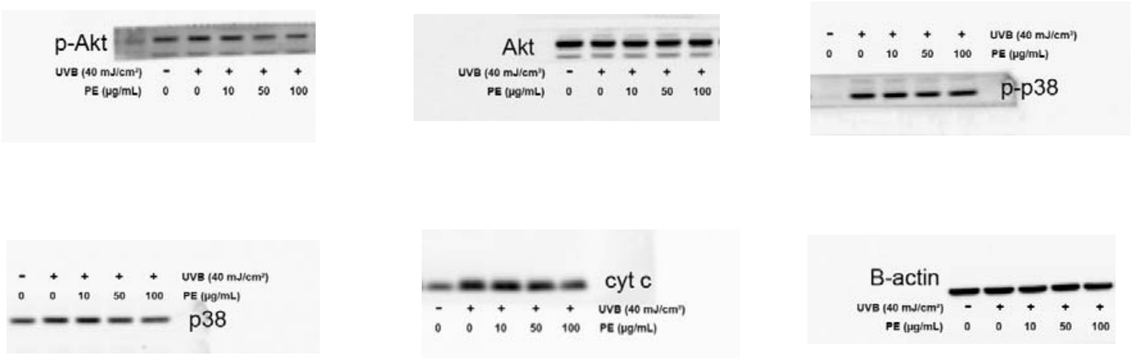

Figure S5. Original images of western blot band intensities in Figure 6.
